# Quantitative evaluation of the effect of anatomical changes on the results of in vivo dosimetry during radiation therapy in the thoracic region

**DOI:** 10.1002/acm2.70542

**Published:** 2026-03-25

**Authors:** Kaname Tanaka, Masataka Komori

**Affiliations:** ^1^ Department of Radiation Therapy Toyota Memorial Hospital Toyota Aichi Japan; ^2^ Department of Integrated Health Sciences Nagoya University Graduate School of Medicine Nagoya Aichi Japan

**Keywords:** anatomical changes, electronic portal imaging device, gamma passing rate, in vivo dosimetry, intensity‐modulated radiation therapy, volumetric modulated arc therapy

## Abstract

**Background:**

Anatomical changes during thoracic radiation therapy can alter dose delivery and compromise accuracy. Electronic portal imaging device (EPID)‐based in vivo dosimetry (IVD) enables noninvasive monitoring of these changes during treatment.

**Purpose:**

This study quantitatively evaluated the impact of EPID‐based IVD in volumetric modulated arc therapy (VMAT) and explored the potential of IVD for guiding timely replanning.

**Methods:**

Eleven patients undergoing thoracic VMAT were retrospectively analyzed: six required replanning as a result of anatomical changes (replanning group), and five did not (normal group). For each fraction, cumulative EPID transit images were acquired, and the global gamma passing rate (GPR) was calculated using six criteria. The difference between treatment GPR and patient‐specific QA GPR (ΔGPR) was used to reduce plan‐specific bias. For four patients in the replanning group (excluding two patients who were excluded due to changes in treatment machines or immobilization), the original plans were recalculated on post‐replanning CT images to estimate dose changes in the planning target volume (PTV) and organs at risks (OARs).

**Results:**

Across all criteria, the normal group demonstrated higher GPR and ΔGPR values. The 3%/3 mm criterion best discriminated between groups, with a per‐fraction GPR of 98.2% ± 1.1% (normal) versus 90.5% ± 7.1% (replanning). GPR improved after replanning but remained below the levels observed in the normal group. Dose simulations indicated that continuing treatment without replanning increased the PTV dose (D50% up to + 3.5%) and OAR doses, with increases exceeding 5 Gy in some cases.

**Conclusions:**

EPID‐based IVD detected reduced GPR values in replanning cases, identifying the 3%/3 mm criterion as optimal. A per‐fraction GPR of < 97% under this criterion may indicate notable anatomical changes. Continuing treatment without replanning tended to increase doses delivered to the target and normal tissues. These findings support the clinical utility of IVD and provide quantitative criteria for replanning decisions beyond CT or contour‐based assessments.

## 1 INTRODUCTION

Patients often experience anatomical changes during thoracic radiation therapy.^1^ Tumor shrinkage and variations in pleural effusion are common.^2^ Treatment plans are typically based on the initial computed tomography (CT) scan. When anatomical changes occur, discrepancies can arise between the calculated and delivered doses, increasing the risk of overdosing and underdosing.

Intensity‐modulated radiation therapy (IMRT) achieves a steep dose gradient; however, its precision may be compromised by anatomical changes.^3^ Image‐guided radiotherapy (IGRT) can detect such changes immediately before irradiation.^4^ Nevertheless, it remains difficult to evaluate dose delivery quantitatively, and its ability to guide replanning decisions is limited.^1^, ^5^ Frequent CT imaging also raises concerns regarding additional radiation exposure.^6^

Radiation oncology professionals must improve treatment accuracy and ensure patient safety. With the widespread use of high‐precision techniques such as IMRT and stereotactic radiotherapy, accurate dose management has become essential. In vivo dosimetry (IVD) has attracted attention as a method for directly verifying whether radiation is delivered according to the treatment plan during each fraction.^7^, ^8^, ^9^, ^10^, ^11^

Conventional IVD methods use physical dosimeters, such as metal‐oxide‐semiconductor field‐effect transistors (MOSFETs), placed at the irradiation site. These methods face several challenges, including complex setup procedures, limited measurement ranges, and a lack of real‐time feedback. Recently, IVD techniques utilizing transit images acquired with amorphous‐silicon–based electronic portal imaging devices (EPIDs) have emerged to overcome these limitations.^12^, ^13^, ^14^, ^15^, ^16^

EPIDs were initially designed for portal image acquisition during treatment. Their high spatial resolution and stable dose–response characteristics allow them to function as noninvasive, real‐time dosimeters capable of capturing two‐dimensional dose distributions.^17^ EPID‐based IVD offers multiple clinical benefits, including verification of dose delivery accuracy, detection of patient misalignment, and early identification of equipment malfunctions.^18^

The American Association of Physicists in Medicine (AAPM) Task Group 307 has extensively reported on EPID‐based IVD models, algorithms, and clinical experience.^10^ IVD measures radiation dose during treatment to detect deviations arising from equipment malfunctions, dose‐calculation errors, patient misalignment, or anatomical changes. The AAPM TG‑307 classifies EPID‑based IVD approaches into zero‑dimensional (0D), two‑dimensional (2D), and three‑dimensional (3D) methods, with 2D techniques analyzing EPID‑acquired dose distributions via forward‐ or back‑projection approaches. While 3D IVD enables volumetric dose reconstruction and dose–volume histogram (DVH) analysis, it requires high computational resources, increased cost, and precise anatomical registration. Owing to its simplicity, workflow compatibility, and ability to rapidly detect mechanical or patient‑related errors, 2D IVD is widely used and remains a practical tool for daily clinical application in high‑precision radiotherapy. Celi et al. analyzed 2 years of IVD data and demonstrated that IVD improved treatment reliability and safety.^16^

Many studies have addressed anatomical changes in head and neck radiotherapy^19^, ^20^, ^21^; however, reports focusing on the thoracic region remain limited.^1^, ^2^, ^22^ Peca et al. evaluated IVD accuracy in low‐density lung regions,^23^ whereas Cilla et al. reported IVD results during stereotactic radiotherapy for metastatic lung cancer.^22^ Feng et al. investigated the anatomical changes using daily CT images and developed a novel IVD‐based dose calculation method for lung cancer.^24^

To date, the relationship between anatomical changes in the thoracic region and measured IVD results remains unclear. In IMRT, objective methods and standardized criteria for guiding replanning decisions have not yet been established.

This study aimed to quantitatively evaluate the impact of anatomical changes on IVD results during thoracic radiation therapy. This analysis sought to support earlier and more appropriate planning decisions. By using measured dose data transmitted through the body, radiation oncology professionals may objectively determine the timing of replanning and avoid overdosing and underdosing.

## 2 METHODS

### 2.1 Patient selection

This study included 11 patients who underwent thoracic radiation therapy using volumetric modulated arc therapy (VMAT)^25^, ^26^ during free breathing. Superficial cases, such as breast cancer, were excluded. The cohort was divided into two groups: six patients who required replanning as a result of anatomical changes (replanning group) and five patients who did not require replanning and exhibited no anatomical changes (normal group). All patients were treated with 6 MV X‐rays. For image‐guided positioning, the ExacTrac system (Brainlab AG, Munich, Germany) was used to perform six‐axis corrections based on orthogonal X‐ray images, followed by target alignment using cone‐beam computed tomography (CBCT). Patients who underwent pre‐scheduled replanning were excluded from the study, as were those whose treatment plans were predetermined to change after 20 of the 30 fractions. In the control (normal) group, clinical staff visually compared daily CBCT images with the initial CT simulation images. Patients showing any suspected anatomical changes, identified by comparing target contours, lung anatomy, or body surface, were excluded from the normal group.

### 2.2 EPID‐based IVD

**FIGURE 1:**
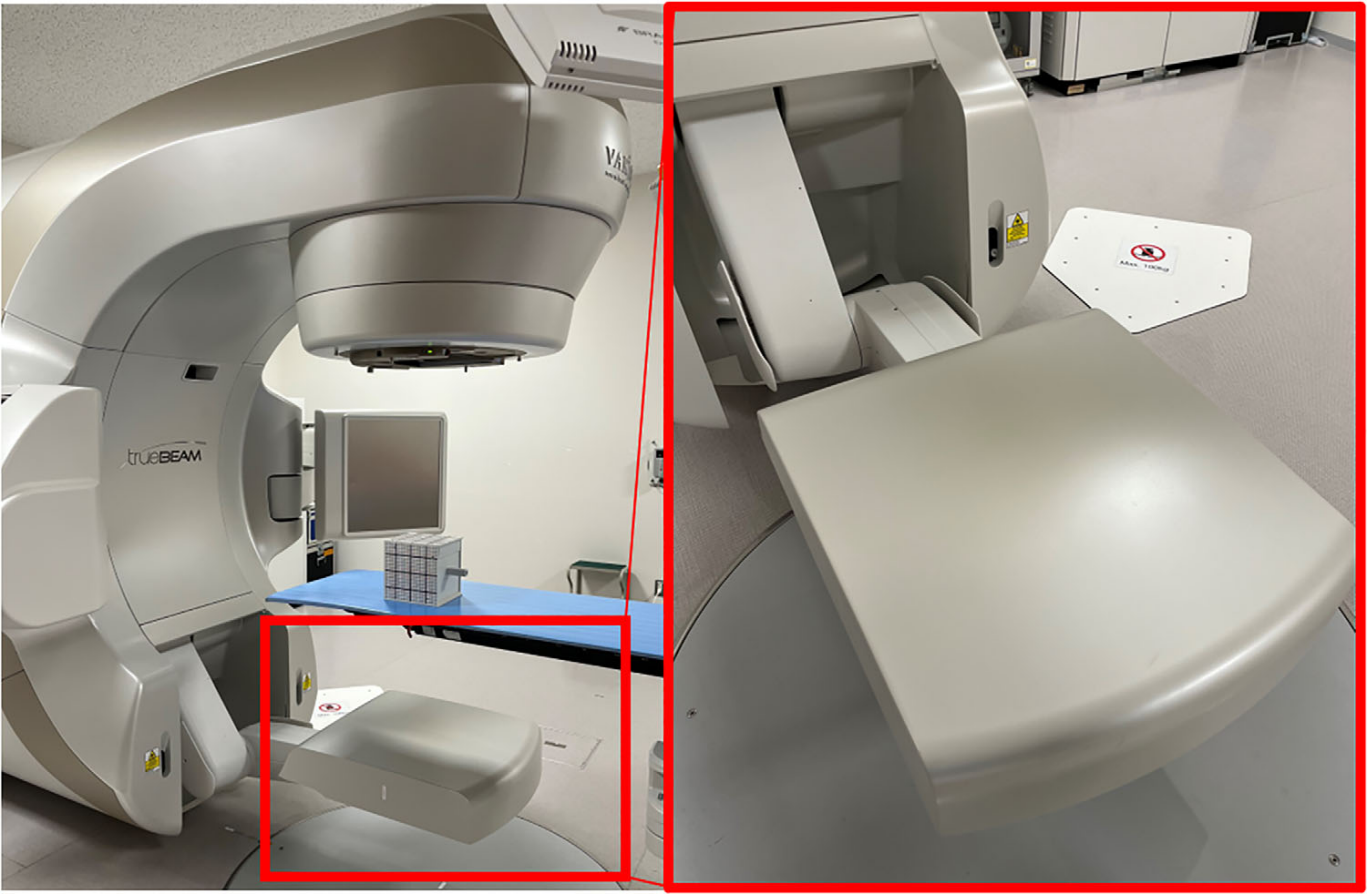
External view of the TrueBeam linear accelerator illustrating the positional relationship and relative size of the gantry‐mounted EPID. The EPID, based on an amorphous‑silicon detector panel, records treatment beams transmitted through the patient, providing measured data for IVD.

**FIGURE 2:**
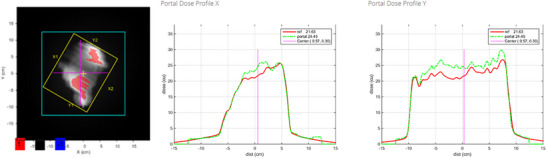
Analysis performed using ADAPTIVO software, comparing cumulative dose distribution data measured with the EPID to dose distributions calculated by the TPS. In the figure, EPID‐measured distributions are indicated by green dashed lines, whereas TPS‐calculated distributions are indicated by red solid lines.

Radiation treatments were delivered using the TrueBeam 2.5 system (Varian Medical Systems, Palo Alto, CA, USA), and treatment planning was performed using Eclipse v13.7 (Varian). All treatment plans included target contours delineated by radiation oncologists. Three gross tumor volumes (GTVs) were individually defined using free‐breathing–like inspiratory breath‐hold phase plain CT images, free‐breathing–like expiratory breath‐hold phase plain CT images, and slow‐scan plain CT images, which served as reference images. The combined volume of these GTVs was defined as the “GTV Sum.” Subsequently, a clinical target volume (CTV) was defined by expanding the GTV Sum as appropriate for each case. A planning target volume (PTV) was then defined by adding a 5 mm isotropic margin to the CTV. The EPID used was the a‐Si 1200 model (Varian), with a resolution of 1190 × 1190 pixels, a pixel pitch of 0.336 mm, and an effective field size of 40 × 40 cm^2^ (Figure 1). Using this setup, cumulative EPID dose measurements were acquired for each treatment field in every fraction for all patients. Fractions in which treatment was interrupted owing to equipment malfunction, resulting in split dose measurements across multiple fields, were excluded from analysis. A total of 274 EPID transit images were analyzed using Adaptivo software (Standard Imaging, Middleton, WI, USA) (Figure 2). The software automatically excluded dose contributions outside the primary collimator aperture. Dose prediction was performed by first removing the flood field applied by the Varian system from the measured image and subsequently applying a portal sensitivity map, enabling off‐axis movement of the EPID when necessary. The resulting predicted image represents the inherent EPID response to the treatment beam, including energy dependence and backscatter from the EPID arm, with the backscatter component corrected using an empirical method. The prediction algorithm models the EPID response by using a fluence matrix derived from multileaf collimator (MLC) control points, convolved with energy‐dependent dose‐deposition kernels. Off‐axis fluence profiles and beam softening effects are also included in the model. For analysis, data from the initial fraction and every fifth fraction were extracted for each patient. Gamma analysis was performed within the software to compare the measured data with the calculated data using global dose normalization, with the percentage dose difference criterion defined relative to the maximum dose in the reference distribution. In the ADAPTIVO software, the normalization value was fixed by design and cannot be modified by the user.

### 2.3 Data analysis and evaluation

A retrospective analysis of IVD results was performed for all patients. The IVD results reflect not only patient‐related anatomical changes but also irradiation errors originating from the treatment delivery system, such as output variations and MLC positional deviations. Although treatment machines undergo routine quality assurance (QA) by medical physicists, residual errors within acceptable tolerance levels remain and cannot be completely eliminated. Furthermore, treatment plans are tailored for each patient, with variations in target size, irradiation field extent, total monitor units, and the degree of intensity modulation. Plans with steep dose gradients within the irradiation field may exhibit larger errors in IVD results compared to relatively flat dose distributions. Patient‐specific quality assurance (PSQA) using EPIDs can detect equipment‐related and plan‐specific errors in the absence of the patient. To minimize the influence of treatment machine performance and plan‐related factors, the difference between the gamma passing rate (GPR) measured during actual treatment delivery and that obtained during PSQA was defined as ΔGPR, and both GPR and ΔGPR were used for evaluation. Six thresholds were tested to determine the optimal GPR evaluation criteria: 1%/1 mm, 2%/2 mm, 3%/2 mm, 3%/3 mm, 4%/4 mm, and 5%/5 mm. Criteria that are too strict demand a level of precision beyond the daily accuracy maintained by equipment quality control, resulting in decreased pass rates even in the normal group and making it difficult to distinguish from the replanning group. Conversely, overly permissive criteria cause all results to approach 100%, preventing accurate evaluation. Therefore, radiation therapy facilities need to consider appropriate criteria according to their specific purposes. The AAPM recommends, for IMRT PSQA, a dose threshold of ≥10% of the prescription dose, a dose difference (DD) of 3%, and a distance‐to‐agreement (DTA) of 2 mm. However, the most commonly used criterion, 3%/3 mm, remains valuable for interinstitutional comparisons. Continued use of this criterion for EPID‐based QA has also been reported. Based on these findings, six GPR criteria (1%/1 mm, 2%/2 mm, 3%/2 mm, 3%/3 mm, 4%/4 mm, and 5%/5 mm) were applied to compare data across all patients. The above results were categorized into four data groups: PSQA, the replanning group before replanning, the normal group, and the replanning group after replanning. The category “the replanning group after replanning” represents the comparison between the predicted images of the replanned treatment plan (New Plan) and the EPID images acquired during irradiation with this New Plan.

### 2.4 Dose distribution estimation in patients with anatomical changes

**FIGURE 3:**
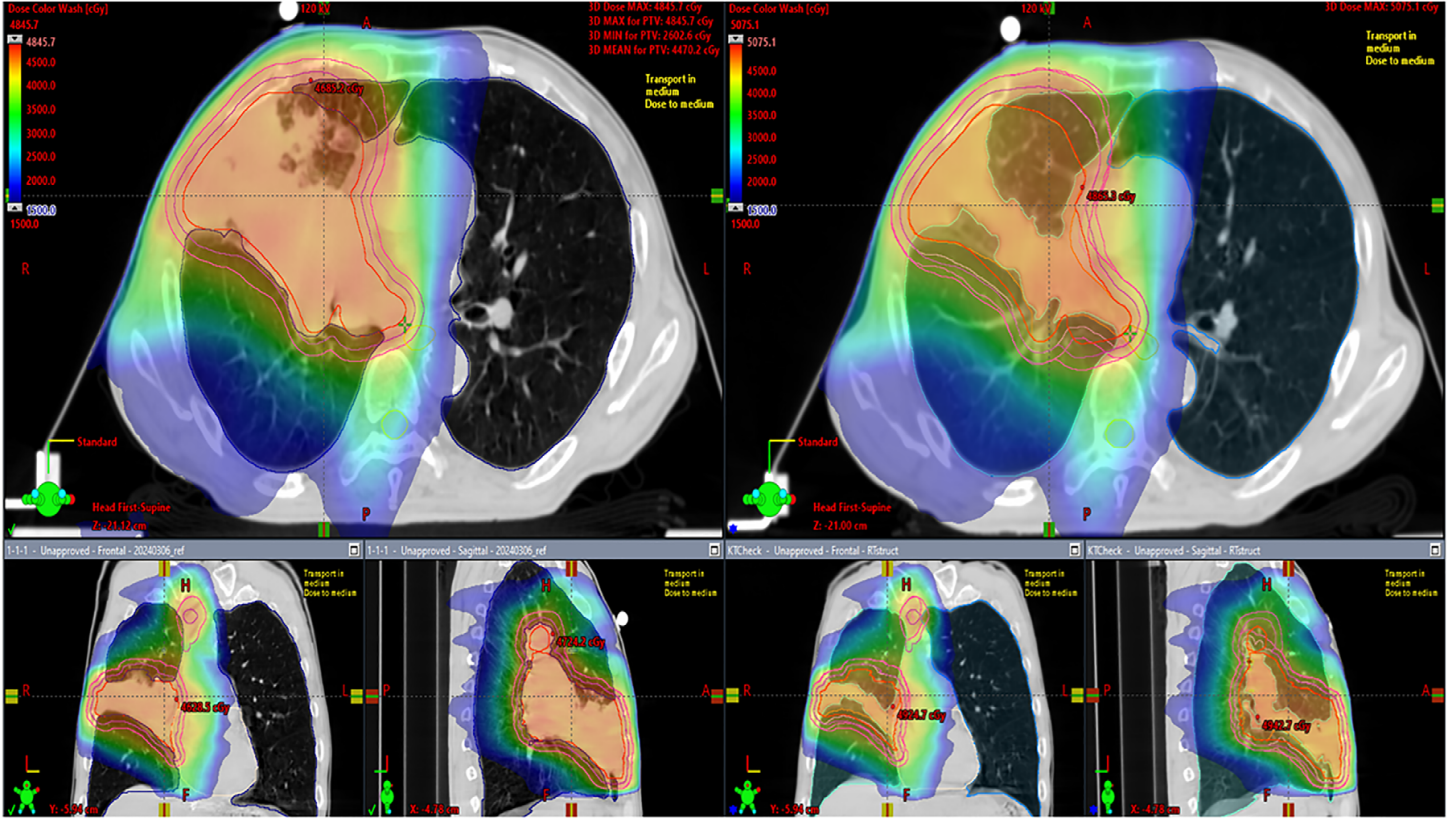
Dose‐distribution assessment in the replanning group. The original treatment plan was applied to a newly acquired CT dataset and recalculated in the TPS, followed by DVH evaluation for the target and OARs. To model treatment continuation under anatomical changes, target contours from the original plan were used, whereas OAR contours from the replanned dataset were adopted.

To evaluate the impact of anatomical changes on dose distribution, the original treatment plans (Original Plan) were recalculated on post‐replanning CT images (New CT) using the treatment planning system (TPS) (Figure 3). By performing dose recalculation without optimization in the TPS, this analysis simulated the effect of anatomical changes on the DVH under the scenario where the Original Plan would have been used to continue treatment without replanning. Two of the six patients in the replanning group were excluded due to changes in treatment machines or immobilization devices at the time of replanning; consequently, the analysis included the remaining four patients. The original target contours were used to simulate continued treatment without replanning, whereas the normal tissue contours were updated to reflect anatomical changes. Dose metrics for the PTV, D2%, D50%, and D98%, were evaluated before and after replanning. For normal tissues, the dose constraints proposed by Timmerman et al., which are routinely used in clinical practice, were applied to assess dose changes.

## 3 RESULTS

### 3.1 EPID‐based IVD measurement and analysis results

Table 1 summarizes the GPR and ΔGPR values for the four data categories. As expected, stricter evaluation criteria yielded lower GPR values, whereas more lenient criteria produced higher GPRs. Correspondingly, the absolute values of ΔGPR decreased as the criteria became less stringent. For clarity, a higher GPR indicates a greater agreement between the measured dose distribution during treatment and the planned dose distribution. In gamma analysis, the evaluation criteria combine a DD threshold and a DTA threshold. When these thresholds are more stringent—that is, allowing only very small differences in dose or position—the test becomes harder to pass, resulting in lower GPR values. Conversely, less stringent criteria permit larger differences, making agreement easier to achieve and yielding higher GPR values. Among the six GPR criteria applied, representative results for the 3%/2 mm and 3%/3 mm criteria are presented, as these were deemed appropriate based on the results shown in Table 1. Under the 3%/3 mm criterion, the GPRs for the replanning and normal groups were 90.5 ± 7.1% and 98.2 ± 1.1%, respectively. Under the 3%/2 mm criterion, the corresponding GPRs were 87.4 ± 7.9% and 96.1 ± 1.7%, respectively. Because ΔGPR for PSQA is defined as the difference between the PSQA GPR and itself, its value was consistently zero across all evaluation criteria. Across all evaluation conditions, the normal group demonstrated superior GPR and ΔGPR values compared with the replanning group. In the replanning group, GPR and ΔGPR improved after replanning, indicating enhanced dose‐delivery accuracy following anatomical adjustments.

**TABLE 1:** GPR and ΔGPR from EPID‐based IVD in four groups.

		PSQA^a^			Replanning group			Normal group			After replanning		
Criteria		Ave		SD	Ave		SD	Ave		SD	Ave		SD
1%/1 mm	GPR (%)^b^	70.4	±	6.0	56.4	±	13.1	56.4	±	13.1	58.1	±	14.2
	ΔGPR	0.0	±	0.0	−13.9	±	11.1	−10.0	±	7.1	−12.9	±	14.0
2%/2 mm	GPR (%)	94.7	±	3.6	81.5	±	10.1	91.0	±	3.7	84.2	±	10.7
	ΔGPR	0.0	±	0.0	−14.2	±	10.4	−1.9	±	3.8	−11.5	±	10.9
3%/2 mm	GPR (%)	*97.7*	±	1.7	87.4	±	7.9	96.1	±	1.7	89.6	±	9.1
	ΔGPR	0.0	±	0.0	−10.7	±	8.1	−0.8	±	2.2	−8.7	±	9.0
3%/3 mm	GPR (%)	*99.6*	±	0.4	90.5	±	7.1	98.2	±	1.1	92.2	±	7.9
	ΔGPR	0.0	±	0.0	−9.2	±	7.2	−1.1	±	0.9	−7.7	±	7.9
4%/4 mm	GPR (%)	*100.0*	±	0.0	95.1	±	4.5	99.4	±	0.6	97.0	±	3.7
	ΔGPR	0.0	±	0.0	−4.8	±	4.5	−1.3	±	0.6	−3.0	±	3.7
5%/5 mm	GPR (%)	*100.0*	±	0.0	97.4	±	3.0	99.8	±	0.2	98.4	±	2.0
	ΔGPR	0.0	±	0.0	−2.5	±	3.0	−0.2	±	0.2	−1.6	±	2.0

*Note*: Gamma Passing Rate (GPR) and ΔGPR obtained from EPID‐based IVD for four patient groups: patient‐specific quality assurance (PSQA), replanning group before replanning, anatomically stable normal group, and replanning group after replanning. GPR was calculated under six evaluation criteria (1%/1 mm to 5%/5 mm) with global dose normalization, and the results are expressed as mean ± standard deviation. ΔGPR was defined as the difference between treatment GPR and PSQA GPR to eliminate plan‐specific bias.

Abbreviations: Ave, average; SD, standard deviation.

^a^ Patient‐specific quality assurance (PSQA).

^b^ Gamma Passing Rate (GPR).

### 3.2 Boxplot analysis of GPR and ΔGPR

**FIGURE 4:**
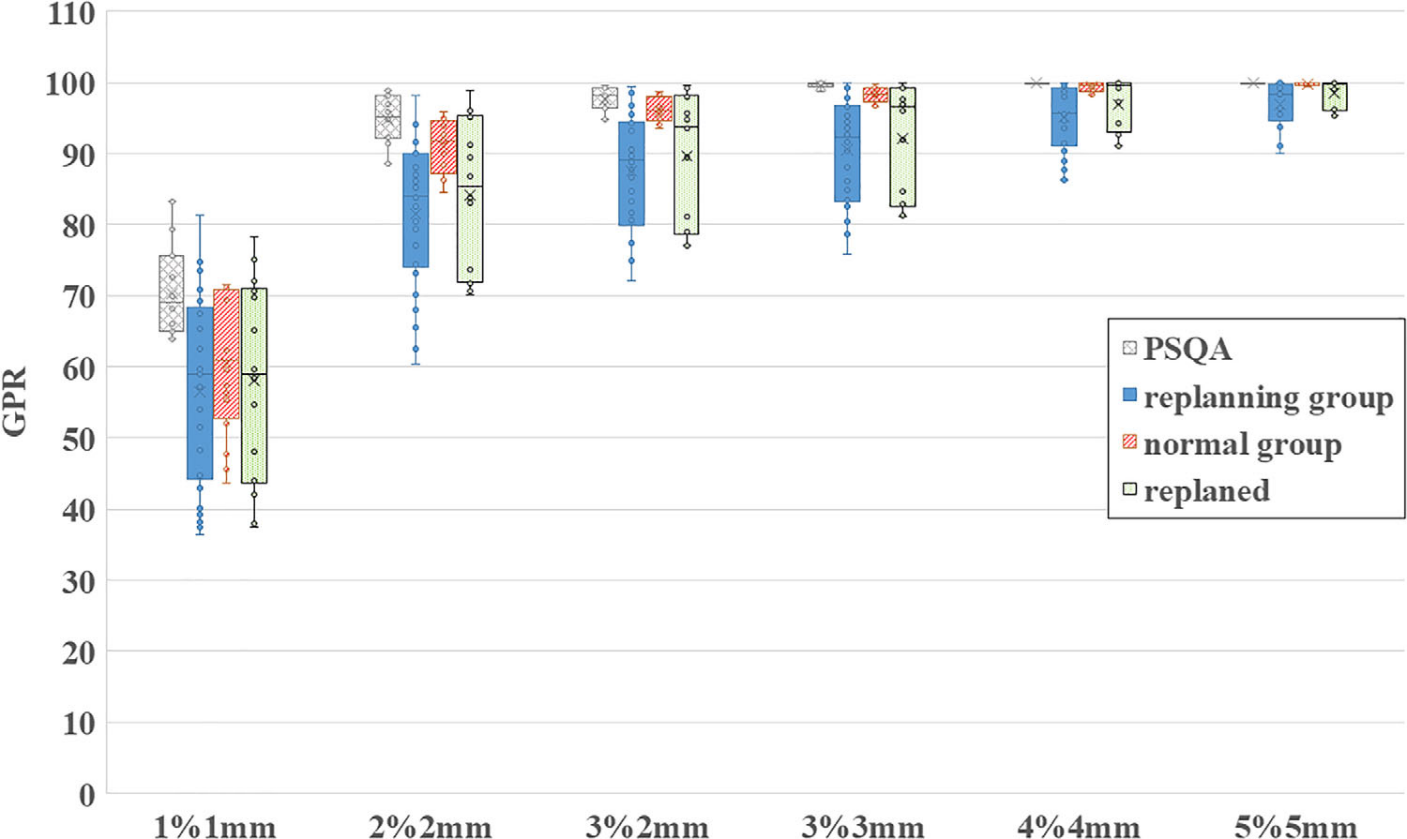
GPR from IVD under six evaluation criteria. Vertical axis: GPR (%) with global dose normalization; horizontal axis: criteria from stringent (left) to lenient (right). Gray = patient‑specific QA (PSQA); blue = replanning group before replanning; red = anatomically stable normal group; green = replanning group after replanning. PSQA reached 100% under lenient criteria, masking plan differences. Post‐replanning GPR (green) improved compared with pre‐replanning (blue) but remained below the normal group (red) across all criteria. Overall, the replanning group exhibited consistently lower GPR values than the normal group, whereas the PSQA group yielded the highest values.

**FIGURE 5:**
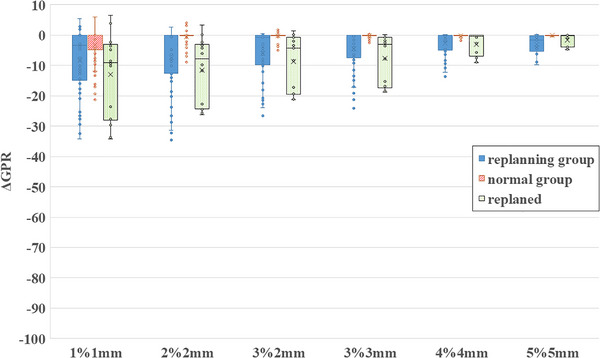
ΔGPR calculated by subtracting patient‑specific QA (PSQA) GPR from IVD GPR, removing plan‑dependent effects and enabling clearer dose assessment. Blue = replanning group before replanning; red = anatomically stable normal group; green = replanning group after replanning. Horizontal axis: GPR criteria from stringent (left) to lenient (right); PSQA data not shown. Variability decreased under lenient criteria, whereas criteria stricter than 3%/3 mm occasionally yielded positive ΔGPR as a result of statistical effects. Most normal‑group data clustered near zero, indicating minimal anatomical change and allowing evaluation of other factors such as setup variation.

**FIGURE 6:**
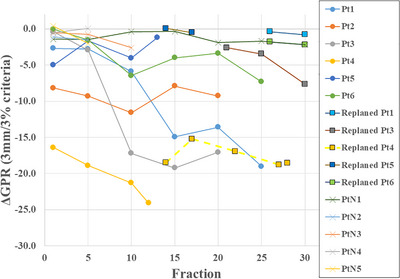
ΔGPR during treatment for individual patients, calculated by subtracting patient‑specific QA (PSQA) GPR from IVD GPR to remove plan‑dependent effects. Solid circles = replanning group; solid squares = anatomically stable normal group; dashed lines with crosses = post‐replanning data. Horizontal axis: treatment fraction; vertical axis: ΔGPR. The replanning group generally showed lower ΔGPR values than the normal group, often declining over time, with some reductions from the first fraction. Post‐replanning values improved but remained below the normal‐group levels.

Figure 4 presents the boxplots of GPR values across all evaluation criteria. Distinct visual markers were used: solid blue for the replanning group, red diagonal stripes for the normal group, gray crosshatch for PSQA, and green dotted lines for the post‐replanning results. Across all criteria, the replanning group consistently showed lower GPR values than the normal and PSQA groups. Under criteria more lenient than 3%/3 mm, PSQA results reached 100%, limiting their discriminatory value. Figure 5 shows boxplots of ΔGPR values for each evaluation criterion. Under stricter criteria than 3%/3 mm, ΔGPR values occasionally appeared positive. Although GPR during treatment should not exceed PSQA values, excessively stringent criteria can introduce small fluctuations and standard deviations that disproportionately influence results. Based on Table 1, Figure 4, and Figure 5, the 3%/3 mm criterion was determined to be the most effective for distinguishing between the normal and replanning groups. Figure 6 illustrates ΔGPR values per treatment fraction for each patient under the 3%/3 mm criterion. The x‐axis represents the fraction number, and the y‐axis represents GPR. Because GPR was calculated by subtracting PSQA values, the PSQA data are not displayed. Circular markers denote the replanning group, crosses indicate the normal group, and squares represent post‐replanning data. Consistent color schemes were used for pre‐ and post‐replanning data for the same patient. In the replanning group, ΔGPR tended to decrease as treatment progressed, whereas the normal group maintained values close to zero and clustered near the top of the graph. All patients in the replanning group showed improvement after replanning; however, GPR values gradually decreased over time, indicating continuous anatomical changes impacting dose delivery.

### 3.3 Dose distribution simulation using TPS

Using the TPS, the original plans were recalculated on the new CT images of four patients in the replanning group. The results are summarized in Tables 2 and 3. In these tables, the data categories ‘recalculation,’ ‘original,’ and ‘ΔDose’ represent the dose distribution obtained by recalculating the Original Plan on the New CT, the dose distribution of the Original Plan as calculated in the TPS, and the difference between these two values (recalculation minus original), respectively. Table 3 presents the dose metrics for organs at risks (OARs), including the bronchus, esophagus, spinal cord, heart, and lungs. Organs located outside the irradiation field and not defined in the plan are marked as “NA.”

**TABLE 2:** TPS simulation of anatomical change impact on PTV dose (D2%, D50%, and D98%).

		PTV D98%	PTV D50%	PTV D2%
Recalculation	Pt1	82.1	103.4	111.1
	Pt2	96.4	102.7	107.1
	Pt3	90.8	101.5	106.9
	Pt4	90.3	100.3	103.2
Original	Pt1	62.8	99.9	103.5
	Pt2	94.9	100.0	102.6
	Pt3	90.5	100.0	103.4
	Pt4	90.7	100.0	102.4
ΔDose	Pt1	19.3	3.5	7.6
	Pt2	1.5	2.7	4.5
	Pt3	0.3	1.5	3.5
	Pt4	−0.5	0.3	0.9

*Note*: Recalculation, The original treatment plans (Original Plan) were recalculated on post‐replanning CT images (New CT) using the treatment planning system (TPS). Original, “Original” indicates the dose distribution of the Original Plan as calculated in the TPS. ΔDose, “ΔDose” indicates the value obtained by subtracting the Original data from the Recalculation data.

Abbreviations: Pt, patient; PTV, planning target volume.

**TABLE 3:** TPS simulation of anatomical change impact on OAR dose metrics.

		Trachea	Esophagus	Spinal cord	Heart	Lung
		D0.03cc (cGy)				V2000cGy (%)
Recalculation	Pt1	6506.6	6304.5	4514.8	NA	12.3
	Pt2	6326.2	6210.5	4336.4	NA	31.0
	Pt3	4877.0	4799.0	3226.1	4916.3	31.0
	Pt4	5274.8	NA	4306.4	NA	20.1
Original	Pt1	6197.7	6063.7	3948.8	NA	11.5
	Pt2	6209.2	6075.3	4095.6	NA	25.5
	Pt3	4722.6	4617.0	3055.2	4690.8	25.1
	Pt4	5180.2	NA	4261.4	NA	18.7
ΔDose	Pt1	308.9	240.8	566.0	NA	0.8
	Pt2	117.0	135.3	240.8	NA	5.5
	Pt3	154.5	182.0	170.9	225.4	6.0
	Pt4	94.6	NA	44.9	NA	1.4

*Note*: Recalculation, The original treatment plans (Original Plan) were recalculated on post‐replanning CT images (New CT) using the treatment planning system (TPS). Original, “Original” indicates the dose distribution of the Original Plan as calculated in the TPS. ΔDose, “ΔDose” indicates the value obtained by subtracting the Original data from the Recalculation data.

Abbreviations: NA, not available; OAR, organ at risk; Pt, patient.

For the PTV, most dose metrics indicated an increased dose following anatomical changes. Specifically, D50%, a representative metric for the prescribed dose, showed a maximum increase of 3.5%. Only one case showed a decrease in D98%, a surrogate for the minimum dose, ranging from −0.3% to −0.8%, indicating minimal change. All evaluated metrics for normal tissues showed increased absorbed doses. Notably, one case demonstrated an increase exceeding 5 Gy in the spinal cord. The magnitude of dose escalation varied among treatment plans, and cases with greater increases in target dose also tended to show larger increases in the doses delivered to surrounding normal tissues.

## 4 DISCUSSION

The GPR values observed in the normal group in this study were slightly higher than the average GPR of 97.2% ± 2.6% reported by Yedekci et al. for VMAT treatments.^27^ Their cohort included patients with anatomical changes, which supports the validity of our results. In the replanning group, the measured transit images consistently showed higher delivered doses than the TPS‐calculated portal dose predictions. This increase is likely due to photon paths shifting through low‐density regions, such as the lungs, resulting from tumor shrinkage, although the contribution of other factors cannot be entirely excluded. IVD results may also be influenced by errors originating from the treatment equipment or by anatomical structures in the beam path that were absent during planning CT acquisition. Although the combination of IVD with CBCT can help identify some of these causes, this remains a limitation of the method. In this study, we employed a 2D in IVD approach. In contrast, 3D IVD techniques reconstruct volumetric dose distributions by back‑projecting EPID images onto patient CT datasets or phantoms, thereby enabling DVH analysis for targets and OARs and directly relating to clinically relevant dose metrics. Furthermore, 3D IVD can reveal localized dose deviations that may not be detected by planar (2D) evaluation. Despite these advantages, 3D IVD is subject to inherent limitations associated with mismatches between contours defined on the planning CT and those reflecting post‐treatment anatomical changes. While deformable contouring techniques exist, their current accuracy is not yet sufficient to fully address these issues in routine clinical practice, partly due to the associated workload. In addition, 3D IVD typically requires CBCT or other CT imaging, which entails additional radiation exposure and may prolong treatment time per fraction, representing potential disadvantages to the patient. In contrast, 2D IVD does not provide volumetric dose data or DVH analysis, but offers advantages including the absence of additional patient exposure, simplicity, efficiency, and compatibility with existing clinical workflows. In this study, no direct comparison was made with alternative methods such as 3D IVD back‑projection.

Figures 4, 5, 6 revealed that the IVD results improved temporarily after replanning but subsequently declined. Immediately after replanning, the values did not reach the levels observed in the normal group. Based on clinical experience, anatomical changes tend to be gradual in the early treatment phase and more pronounced in later stages; the post‐replanning data likely reflect this trend. In most cases, replanning is performed only once. Toward the end of treatment, radiation oncology professionals often continue with existing plans without further replanning. Consequently, late‐phase data included in the post‐replanning analysis may negatively influence the average GPR values. In thoracic radiotherapy, concurrent chemotherapy often causes weight loss and misalignment with immobilization devices, especially in later stages. These factors may also contribute to unfavorable IVD results.

IVD offers the advantage of acquiring dose data without additional radiation exposure. In cases requiring frequent CBCT imaging to monitor anatomical changes, IVD can help reduce cumulative patient dose. Even in facilities that do not perform CBCT for every fraction, IVD remains a valuable tool. Because IVD evaluation occurs after irradiation, it does not extend treatment time and can be implemented even when CBCT acquisition is limited by the patient's condition or time constraints. In clinical practice, replanning to reduce the irradiation field must be approached with caution because of the risk of tumor recurrence.^28^ However, progressive anatomical changes can distort dose distribution and increase the risk of high‐dose exposure to normal tissues. Balancing these risks is essential. Quantitative evaluation using IVD enables a more precise determination of the need for and timing of replanning. This approach overcomes the limitations of subjective CBCT‐based assessments. In particular, observing a downward trend in the GPR can help identify the optimal timing for intervention. As shown in Figure 6, atypical changes were observed during the initial fractions in several cases. During thoracic treatment, anatomical changes may begin before therapy owing to the effects of chemotherapy.^29^, ^30^ The initial IVD results can serve as a tool to detect such early changes. If a GPR decline is observed early during treatment, radiation oncology professionals can proactively schedule replanning to accommodate evolving anatomy. Traditionally, treatment is continued as long as the target remains within the PTV. However, this approach is risky for IMRT, in which dose heterogeneity and overexposure of normal tissues may occur. IVD provides a quantitative basis for early intervention to mitigate these risks.

Table 2 shows that the target dose generally increased. Minor reductions in the D98% (up to 0.8%) were considered negligible. No decrease in the GTV D98% was observed; instead, the dose either increased or remained stable. Because an insufficient target dose may lead to tumor recurrence, confirming dose escalation is clinically important. The extent of dose increase varied by case and was likely influenced by the target volume and beam paths through lung tissue. Table 3 shows the dose increases in all normal tissues. Evaluations based on updated contours yielded valuable insights by focusing on cases that underwent replanning. High‐dose changes may be inferred from imaging; however, low‐dose variations, such as lung V20 Gy, are difficult to assess visually. This simulation confirmed violations of dose constraints, highlighting the relevance of anatomical changes and the effectiveness of replanning. Although daily TPS recalculations are impractical in clinical settings, combining TPS with IVD allows for rapid estimation of dose impact.

Figures 4, 5, 6 shows that VMAT plan complexity substantially affected the IVD results. Specifically, according to the principles of gamma analysis, plans with steep dose gradients within the irradiation field tend to yield lower GPR than plans with more uniform dose distributions. This inherent limitation means that highly modulated or complex VMAT plans are more likely to show reduced passing rates, even when dose delivery is accurate. To account for this, PSQA results were subtracted during analysis. Because the treatment site and technique also influence outcomes, this study focused specifically on thoracic VMAT cases. Reports on adaptive radiotherapy have emphasized the need for personalized treatment strategies.^5^ The standardized IVD evaluation metrics proposed by Dogan et al. are effective for detecting major errors.^10^ However, these methods cannot identify subtle changes or trends. Continuous tracking of patient‐specific IVD results is therefore essential. Early detection of deterioration enables timely adjustments, such as increasing CBCT frequency or addressing immobilization mismatches caused by weight loss. Although this study did not fully account for non‐plan‐related factors, future investigations should address variables such as body‐weight changes, physique, setup errors, organ motion, and target‐volume dynamics. CBCT is widely adopted as a standard IGRT tool; however, IVD remains underutilized in many institutions. CBCT and IVD have distinct advantages, and their combined use could enhance the safety and precision of daily radiation therapy. Overall, this study supports the broader adoption of IVD as a standard clinical practice.

## 5 CONCLUSION

This study analyzed the EPID‐based IVD results from clinical VMAT cases. Patients who underwent replanning because of anatomical changes showed decreased GPR values. Among the evaluated criteria, the 3%/3 mm threshold was found to be the most appropriate. Under this criterion, the GPRs were 98.2% ± 1.1% for the normal group and 90.5% ± 7.1% for the replanning group, with the latter showing markedly lower values. Cases falling below 97% under the same criteria may require careful attention for potential anatomical changes. Dose‐distribution simulations for patients who underwent replanning revealed a tendency for increased dose delivery to target volumes and normal tissues when treatment was continued without replanning. These results highlight the importance of timely intervention and support the clinical utility of IVD in guiding adaptive radiotherapy decisions.

## AUTHOR CONTRIBUTIONS

Kaname Tanaka contributed to the design and implementation of the research, to the analysis of the results and to the writing of the manuscript. Masataka Komori contributed to the design and implementation of the research, to the analysis of the results and supervised this study.

## CONFLICT OF INTEREST STATEMENT

The authors declare no conflicts of interest.

## DATA AVAILABITY STATEMENT

Data supporting the findings of this study are available from the corresponding author upon reasonable request.

## References

[1] Kupelian PA , Ramsey C , Meeks SL , et al. Serial megavoltage CT imaging during external beam radiotherapy for non‐small‐cell lung cancer: observations on tumor regression during treatment. Int J Radiat Oncol Biol Phys. 2005;63(4):1024‐1028. doi:10.1016/j.ijrobp.2005.04.046 16005575 10.1016/j.ijrobp.2005.04.046

[2] Erridge SC , Seppenwoolde Y , Muller SH , et al. Portal imaging to assess set‐up errors, tumor motion and tumor shrinkage during conformal radiotherapy of non‐small cell lung cancer. Radiother Oncol. 2003;66(1):75‐85. doi:10.1016/S0167‐8140(02)00287‐6 12559524 10.1016/s0167-8140(02)00287-6

[3] Korreman SS . Motion in radiotherapy: photon therapy. Phys Med Biol. 2012;57(23):R161‐R191. doi:10.1088/0031‐9155/57/23/R161 23165229 10.1088/0031-9155/57/23/R161

[4] Kilburn JM , Soike MH , Lucas JT , et al. Image guided radiation therapy may result in improved local control in locally advanced lung cancer patients. Pract Radiat Oncol. 2016;6(3):e73‐e80. doi:10.1016/j.prro.2015.10.004 26725964 10.1016/j.prro.2015.10.004 PMC5555210

[5] Sonke JJ , Aznar M , Rasch C . Adaptive radiotherapy for anatomical changes. Semin Radiat Oncol. 2019;29(3):245‐257. doi:10.1016/j.semradonc.2019.02.007 31027642 10.1016/j.semradonc.2019.02.007

[6] Ding GX , Alaei P , Curran B , et al. Image guidance doses delivered during radiotherapy: quantification, management, and reduction: report of the AAPM therapy physics committee task group 180. Med Phys. 2018;45(5):e84‐e99. doi:10.1002/mp.12824 29468678 10.1002/mp.12824

[7] The Royal College of Radiologists; Society and College of Radiographers; Institute of Physics and Engineering in Medicine; British Institute of Radiology. Implementing in vivo dosimetry. London: The Royal College of Radiologists; 2008.

[8] Mijnheer B , Beddar S , Izewska J , Reft C . In vivo dosimetry in external beam radiotherapy. Med Phys. 2013;40(7):070903. doi:10.1118/1.4811216 23822404 10.1118/1.4811216

[9] Van Dam J , Marinello G . *ESTRO Booklet No. 1*: Methods for *I*n *V*ivo *D*osimetry in *E*xternal *R*adiotherapy (1st ed. 1994; 2nd ed. 2006). Brussels: European Society for Radiotherapy and Oncology; 2006.

[10] Dogan N , Mijnheer BJ , Padgett K , et al. AAPM task group report 307: use of EPIDs for patient‐specific IMRT and VMAT QA. Med Phys. 2023;50(8):e865‐e903. doi:10.1002/mp.16536 37384416 10.1002/mp.16536 PMC11230298

[11] Olaciregui‐Ruiz I , Beddar S , Greer P , et al. In vivo dosimetry in external beam photon radiotherapy: requirements and future directions for research, development, and clinical practice. Phys Imaging Radiat Oncol. 2020;15:108‐116. doi:10.1016/j.phro.2020.08.003 33458335 10.1016/j.phro.2020.08.003 PMC7807612

[12] Howell RM , Smith IPN , Jarrio CS . Establishing action levels for EPID‐based QA for IMRT. J Appl Clin Med Phys. 2008;9(3):16‐25. doi:10.1120/jacmp.v9i3.2721 18716584 10.1120/jacmp.v9i3.2721 PMC5722294

[13] Miri N , Keller P , Zwan BJ , Greer P . EPID‐based dosimetry to verify IMRT planar dose distribution for the aS1200 EPID and FFF beams. J Appl Clin Med Phys. 2016;17(6):292‐304. doi:10.1120/jacmp.v17i6.6336 27929502 10.1120/jacmp.v17i6.6336 PMC5690494

[14] Kang S , Li J , Ma J , et al. Evaluation of interfraction setup variations for postmastectomy radiation therapy using EPID‐based in vivo dosimetry. J Appl Clin Med Phys. 2019;20(10):43‐52. doi:10.1002/acm2.12712 10.1002/acm2.12712 PMC6806484 31541537

[15] Esposito M , Piermattei A , Bresciani S , et al. Improving dose delivery accuracy with EPID in vivo dosimetry: results from a multicenter study. Strahlenther Onkol. 2021;197(7):633‐643. doi:10.1007/s00066‐021‐01749‐6 33594471 10.1007/s00066-021-01749-6

[16] Celi S , Costa E , Wessels C , Mazal A , Fourquet A , Francois P . EPID based in vivo dosimetry system: clinical experience and results. J Appl Clin Med Phys. 2016;17(3):262‐276. doi:10.1120/jacmp.v17i3.6070 10.1120/jacmp.v17i3.6070 PMC5690938 27167283

[17] Olch AJ , O'Meara K , Wong KK . First report of the clinical use of a commercial automated system for daily patient QA using EPID exit images. Adv Radiat Oncol. 2019;4(4):722‐728. doi:10.1016/j.adro.2019.04.001 31681865 10.1016/j.adro.2019.04.001 PMC6817722

[18] Moustakis C , Ebrahimi Tazehmahalleh F , Elsayad K , Fezeu F , Scobioala S . A novel approach to SBRT patient quality assurance using EPID‐based real‐time transit dosimetry: a step to QA with in vivo EPID dosimetry. Strahlenther Onkol. 2020;196(2):182‐192. doi:10.1007/s00066‐019‐01549‐z 31925465 10.1007/s00066-019-01549-z

[19] Heukelom J , Fuller CD . Head and neck cancer adaptive radiation therapy (ART): conceptual considerations for the informed clinician. Semin Radiat Oncol. 2019;29(3):258‐273. doi:10.1016/j.semradonc.2019.02.008 31027643 10.1016/j.semradonc.2019.02.008 PMC6559245

[20] Castadot P , Lee JA , Geets X , Grégoire V . Adaptive radiotherapy of head and neck cancer. Semin Radiat Oncol. 2010;20(2):84‐93. doi:10.1016/j.semradonc.2009.11.002 20219546 10.1016/j.semradonc.2009.11.002

[21] Matsushita N , Nakamura M , Sasaki M , Yano S , Yoshimura M , Mizowaki T . Analyses of integrated EPID images for on‐treatment quality assurance to account for interfractional variations in volumetric modulated arc therapy. J Appl Clin Med Phys. 2020;21(1):110‐116. doi:10.1002/acm2.12805 31909889 10.1002/acm2.12805 PMC6964755

[22] Cilla S , Ianiro A , Craus M , et al. Epid‐based in vivo dose verification for lung stereotactic treatments delivered with multiple breath‐hold segmented volumetric modulated arc therapy. J Appl Clin Med Phys. 2019;20(3):37‐44. doi:10.1002/acm2.12538 10.1002/acm2.12538 PMC6414179 30790439

[23] Peca S , Brown DW . Two‐dimensional in vivo dose verification using portal imaging and correlation ratios. J Appl Clin Med Phys. 2014;15(4):4752. doi:10.1120/jacmp.v15i4.4752 25207402 10.1120/jacmp.v15i4.4752 PMC5875516

[24] Feng B , Yu L , Mo E , et al. Evaluation of daily CT for EPID‐based transit in vivo dosimetry. Front Oncol. 2021;11:782263. doi:10.3389/fonc.2021.782263 34796120 10.3389/fonc.2021.782263 PMC8592931

[25] Otto K . Volumetric modulated arc therapy: IMRT in a single gantry arc. Med Phys. 2008;35(1):310‐317. doi:10.1118/1.2818738 18293586 10.1118/1.2818738

[26] Li C , Luo H , Song W , Hu Y , Li J , Cai Z . Dosimetric comparison of four radiotherapy techniques for stage III non‐small cell lung cancer. Oncol Lett. 2023;26(2):347. doi:10.3892/ol.2023.13933 37427336 10.3892/ol.2023.13933 PMC10326827

[27] Yedekci Y , Biltekin F , Ozyigit G . Feasibility study of an electronic portal imaging based in vivo dose verification system for prostate stereotactic body radiotherapy. Phys Med. 2019;64:204‐209. doi:10.1016/j.ejmp.2019.07.008 31515021 10.1016/j.ejmp.2019.07.008

[28] Fernandes AT , Shen J , Finlay J , et al. Elective nodal irradiation (ENI) vs. involved field radiotherapy (IFRT) for locally advanced non‐small cell lung cancer (NSCLC): a comparative analysis of toxicities and clinical outcomes. Radiother Oncol. 2010;95(2):178‐184. doi:10.1016/j.radonc.2010.02.007 20356642 10.1016/j.radonc.2010.02.007

[29] Aupérin A , Le Péchoux C , Rolland E , et al. Meta‐analysis of concomitant versus sequential radiochemotherapy in locally advanced non‐small‐cell lung cancer. J Clin Oncol. 2010;28(13):2181‐2190. doi:10.1200/JCO.2009.26.2543 20351327 10.1200/JCO.2009.26.2543

[30] Herskovic A , Martz K , al‐ Sarraf M , et al. Combined chemotherapy and radiotherapy compared with radiotherapy alone in patients with cancer of the esophagus. N Engl J Med. 1992;326(24):1593‐1598. doi:10.1056/NEJM199206113262403 1584260 10.1056/NEJM199206113262403

